# Supplementary material for: Structural mechanisms of pump assembly and drug transport in the AcrAB–TolC efflux system
Source: eLife. 2026 Apr 20;14:RP109684. doi: 10.7554/eLife.109684 (PMC13095208; doi:10.7554/eLife.109684)
Supplement: Supplementary file 1. [file elife-109684-supp1.docx]

**Cryo-EM data collection, refinement and validation statistics**

|  | TolC-YbjP | TolC-YbjP-AcrABZ | TolC-YbjP-AcrA (local) |
| --- | --- | --- | --- |
| Voltage (kV) | 300 | | |
| Electron exposure (e^-^/Å^2^) | 50 | | |
| Defocus range (μm) | -1.2～-1.8 | | |
| Pixel size (Å) | 1.35 | | |
| Micrographs (no.) | 3452 | | |
| Symmetry imposed | C3 | C1 | C3 |
| Final particle images (no.) | 63,022 | 31,000 | 17,043 |
| Map resolution (Å) 0.143 FSC threshold | 3.56 | 3.39 | 3.26 |
| Map sharpening B factor (Å^2^) | -134.5 | -66.6 | -74.7 |
| CC (model vs. data) | 0.7945 | 0.8727 | 0.8456 |
| Model composition |  |  |  |
| Chain count | 6 | 18 | 12 |
| Non-hydrogen atoms | 13,539 | 53,464 | 29,061 |
| Protein residues | 1,752 | 7,037 | 3,816 |
| B factors (Å^2^) |  |  |  |
| Proteins | 40.66 | 99.69 | 115.5 |
| R.m.s. deviations |  |  |  |
| Bond lengths (Å) | 0.006 | 0.003 | 0.006 |
| Bond angles (°) | 0.665 | 0.498 | 0.697 |
| Validation |  |  |  |
| MolProbity score | 2.05 | 1.63 | 1.77 |
| Clashscore | 11.62 | 7.22 | 7.94 |
| Poor rotamers (%) | 0.41 | 0.12 | 0.13 |
| Ramachandran plot |  |  |  |
| Favored (%) | 92.47 | 96.49 | 95.15 |
| Allowed (%) | 7.36 | 3.36 | 4.56 |
| Disallowed (%) | 0.17 | 0.16 | 0.29 |
| PDB code | 9V52 | 9V53 | 9V55 |
| EMDB code | EMD-64784 | EMD-64785 | EMD-64787 |
